# Modeling the Impact of Antiretroviral Use in Developing Countries

**DOI:** 10.1371/journal.pmed.0030148

**Published:** 2006-03-14

**Authors:** Stefano M Bertozzi, Sergio Bautista-Arredondo

## Abstract

Bertozzi and Bautista-Arredondo discuss the implications of a new *PLoS Medicine* study that models the impact of antiretroviral drugs upon HIV transmission in developing countries.

Ideally, evaluations of strategies related to the use of antiretroviral agents would use large-scale randomized trials with HIV-related mortality as the primary endpoint. In reality, however, such studies are rarely conducted, given the long follow-up needed and the substantial costs. Furthermore, people currently living with HIV/AIDS need treatment now, not at the conclusion of long-term prospective trials.

Model-based methods are a powerful and practical means of performing formal comparisons of health interventions when the ideal is not possible. Model-based analyses synthesize data from multiple sources, permitting decision makers to understand the likely impact of different strategies and to set priorities for clinical trials [ 1]. Models also offer a practical framework for managing uncertainty via (1) sensitivity analysis (i.e., sensitivity of the model's outputs to variation in the model's parameters that are either variable or estimated with uncertainty) and (2) analysis of different scenarios (which can represent different settings and/or different levels of optimism in the values of the model's parameters) [ 2, 3]. Finally, models permit analyses that extrapolate beyond the limitations of a trial's time constraints, geographic setting, and study population [ 4, 5].

Probably the most important contribution of mathematical models relies not in the specific point estimates they generate, but rather, in the insights they provide regarding the likely impact of changes in different variables—such as access to treatment, sexual behavior, natural history of the disease, quality of care and adherence—on life expectancy and epidemic dynamics.

## Mathematical Models of HIV Treatment

Mathematical models have been used to assess the impact of antiretroviral programs on HIV transmission [ 6]. Even though there is no consensus on the direction of the effect, many of these studies have predicted that provision of antiretroviral therapy (ART) could have a striking effect on transmission when coverage is high [ 7–10]. However, these studies also suggest that the potential impact of drug-induced biological changes (reduced infectivity or increased duration of infection) on HIV transmission is likely to be minor compared with the potential impact of ART on sexual risk behavior. The impact on sexual behavior is likely to be greater either because availability of treatment potentiates prevention efforts or because it causes disinhibition and increased risk behavior.

Model-based methods are a powerful and practical means of performing formal comparisons of health interventions when the ideal is not possible.

In a study in *PLoS Medicine*, Rebecca Baggaley and colleagues constructed a model that describes the impact of ART on HIV transmission in developing countries, and they chose Malawi to apply their model [ 11]. The authors made a number of assumptions in constructing their model—for example, they assumed that there is only a single, standard triple-combination therapy regimen, with no second line or salvage therapy available for those who experience treatment failure. They used the model to predict and compare the epidemiological impacts of alternative strategies for rolling out ART (for example, initiating treatment at varying stages of disease).

The authors' model predicted that the unlimited provision of ART, started at late-stage infection (AIDS), would lead to an increase in HIV prevalence. The effect of additionally treating patients with earlier infection (pre-AIDS) would depend on whether treated patients changed their sexual behavior. The authors concluded that one cannot expect the provision of ART, at least in high-prevalence African epidemics, to have important effects on HIV transmission, a finding that is consistent with the previous studies summarized above.

## Limitations of the New Model

The authors' modeling was restricted to considering changes in risk behavior by those persons with HIV infection who initiated ART. They did not consider how availability of therapy may lead to dramatic changes in risk behavior (in either direction) among the far larger population of people who are not infected, infected but don't know their status, or infected but not eligible for therapy.

The authors' decision not to consider these effects is understandable given their focus on comparing different initiation/discontinuation strategies for ART, and given the lamentable lack of data on population-level changes in risk behavior following introduction of ART. However, as the work by Mead Over and colleagues in India suggests, small changes in population-level risk behaviors indirectly caused by ART are likely to be far more important determinants of the epidemic's course than are changes among those receiving therapy [ 10]. Thus, we conclude that the key messages regarding ART and prevention seem to be the following: (1) choice of ART initiation, monitoring, and discontinuation strategies is unlikely to have a major impact on prevention, so choose treatment algorithms that maximize treatment efficiency, and (2) focus on how ART implementation can be *prevention-enhancing*. To accomplish the latter, programs must evaluate different strategies to maximize the potential positive impact on prevention and minimize the potential for disinhibition.

The other important limitation of Baggaley and colleagues' study is suggested by the authors in their conclusion: “…in reality, scaling up programmes is likely to compromise quality, meaning higher dropout rates, mortality and treatment failure, negating the beneficial impacts of ART, and increasing the rate of drug resistance emergence.” Not only does the model not capture likely declines in quality with increasing scale, it also does not appear to adequately capture the likely population heterogeneity with respect to quality of care and patient adherence to treatment.

While the model considers four different classes of sexual risk behavior (i.e., four different levels of sexual activity, from high to low), it appears to treat quality and adherence as parameters that are constant across patients and providers. While the amount of bias introduced by this simplification will clearly depend on the true degree of heterogeneity in the population, the highly nonlinear relationship between adherence and viral suppression suggests that the *distribution* of adherence in the population is an important determinant of average effectiveness, not just the mean adherence. Our concern about this issue is heightened because of our experience in Mexico, where we have documented very high levels of provider and patient heterogeneity [ 12]. In poorer countries with fewer drug choices and in those with stronger central control over treatment algorithms (such as Malawi), there is likely to be far less heterogeneity in prescribing practices, but poverty and limited infrastructure may contribute to even greater heterogeneity in drug supply, in patient access to treatment, and in adherence.

## The Policy Implications

Baggaley and colleagues suggest that policy makers, in their quest to maximize the efficiency of treatment, need to consider both the likelihood that a patient will benefit from ART (e.g., probability that they will have immune reconstitution syndrome) and the possibility of reallocating treatment from someone in virological failure to someone who has not yet been able to initiate therapy. If our hypotheses are correct, then it is likely to be even more important, in order to maximize the efficiency of treatment, to consider the probability that a patient will receive care of adequate quality, will have consistent access to drugs, and will be both willing and able to maintain high levels of adherence. The next generation of models should seek to incorporate a more nuanced treatment of these issues—but their ability to do so will also depend on whether data are collected in programs currently being scaled up on prescription patterns, consistency of drug supply, adherence, and duration of viral suppression.

We have argued that mathematical modeling can be very useful for informing policy choices, but it is important that such an argument not be construed to mean that mathematical modeling can replace data on effectiveness of interventions. On the contrary, for future modeling exercises to usefully inform policy, better data are needed. For reasons that are not entirely apparent, the scientific community, nongovernmental organizations, and government officials have not insisted that sufficiently rigorous data be generated on effectiveness of HIV/AIDS interventions as they are scaled up. Without even considering prevention interventions, the amount of information known about how to achieve high rates of ART adherence in developing countries, to take just one example, is nowhere near commensurate with either the size of the problem or the resources being committed to address the problem. If we don't take advantage of the current scale-up of interventions to collect data on which approaches work and which ones don't (thus reducing the depth of our ignorance), it will be impossible to convince people to continue to fund such programs. And if we don't figure out how to implement ART in a way that greatly potentiates the effectiveness of prevention, the costs of care in the future will likely outstrip even the most optimistic projections of resource availability.

## Abbreviation

ART: antiretroviral therapy
